# Strontium- and Zinc-Containing Bioactive Glass and Alginates Scaffolds

**DOI:** 10.3390/bioengineering7010010

**Published:** 2020-01-13

**Authors:** Asfia Haider, Ahmad Waseem, Natalia Karpukhina, Sahar Mohsin

**Affiliations:** 1 Dental Physical Sciences Unit, Institute of Dentistry, Barts & The London School of Medicine and Dentistry, Queen Mary University of London, London E1 4NS, UK; 2 Centre for Immunobiology and Regenerative Medicine, Institute of Dentistry, Barts and The London School of Medicine and Dentistry, London E1 2AD, UK; 3 Department of Anatomy, College of Medicine and Health Sciences, United Arab Emirates University, Al Ain 17666, UAE

**Keywords:** bone scaffolds, alginates, bioactive glass, strontium, zinc, composite

## Abstract

With an increasingly elderly population, there is a proportionate increase in bone injuries requiring hospitalization. Clinicians are increasingly adopting tissue-engineering methods for treatment due to limitations in the use of autogenous and autologous grafts. The aim of this study was to synthesize a novel, bioactive, porous, mechanically stable bone graft substitute/scaffold. Strontium- and zinc-containing bioactive glasses were synthesized and used with varying amounts of alginate to form scaffolds. Differential scanning calorimetric analysis (DSC), FTIR, XRD, and NMR techniques were used for the characterization of scaffolds. SEM confirmed the adequate porous structure of the scaffolds required for osteoconductivity. The incorporation of the bioactive glass with alginate has improved the compressive strength of the scaffolds. The bioactivity of the scaffolds was demonstrated by an increase in the pH of the medium after the immersion of the scaffolds in a Tris/HCl buffer and by the formation of orthophosphate precipitate on scaffolds. The scaffolds were able to release calcium, strontium and zinc ions in the Tris/HCl buffer, which would have a positive impact on osteogenesis if tested in vivo.

## 1. Introduction

Bone is a complex biomineralized system with an intricate hierarchical structure. It is a dynamic structure with a unique capability to renew itself through remodelling. Microdamage occurs in our bones because of everyday cyclical activity. It gets repaired in healthy bone, but it may result in a fragility fracture if the repair mechanism is deficient, as in osteoporotic patients. Large skeletal defects resulting from trauma, malignancies, and various infections are also beyond the capacity of normal bone to repair and often require surgical intervention to restore function. The average yearly number of bone graft surgeries performed worldwide is more than two million, costing $2.5 billion [1,2]. Fractures impose a huge burden on healthcare systems. The combined health and social care costs of hip fractures alone in the UK came to an estimated £1 billion in 2017 [3]. The number of total joint revisions requiring structural bone support and the number of spinal fusions requiring extensive bone graft material is increasing due to an increase in the elderly population. Clinicians are increasingly adopting tissue-engineering methods for treatment to avoid the risk of infection, disease transmission, and histocompatibility differences associated with the use of autografts and allografts [4].

Thus, there is a great need to develop synthetic bone graft substitutes (scaffolds) to meet the epidemiologically driven demand. An ideal bone scaffold should promote early mineralization and support new bone formation while at the same time allowing for replacement by new bone. In order to achieve these goals, the biocompatibility and biodegradability of material is imperative. A scaffold has to have a high interconnected porosity of appropriate size to direct cells to grow into the desired physical form and to support the vascularization of the ingrown tissue [5]. Additionally, it should have a large surface area that promotes cell adhesion, growth, migration, and differentiation. The mechanical properties of the scaffold must be sufficient so that it does not collapse during the patient’s normal activities. Other highly desirable features concerning scaffold processing are its fabrication and scalability for cost-effective industrial production [6].

In this study, alginate and strontium- and zinc-containing bioactive glass was used to create a composite bone scaffold. Alginates have great potential in regenerative medicine because of their biocompatibility, biodegradability, mechanical properties, and relatively low cost [7,8]. Alginate is a polysaccharide obtained from certain species of brown algae, and it is composed of 1,4-linked β-D-mannuronic acid (M) and α-L-guluronic acid (G) residues. The cation and the carboxyl functional groups of G units located on the polymer chain of alginate promote the formation of the hydrogel by the chemical cross-linking of certain divalent cations, such as Ca^2+^ or Sr^2+^ through ionic interaction [7,8]. Alginates have been shown to be able to support the nucleation of hydroxyapatite crystals on their surface when submerged in body fluids and simulated body fluids (SBF) [8]. An SBF solution approximates the ionic constituents, pH, and temperature of blood plasma and has been used widely for in vitro assessment of the bioactivity of various scaffolds.

Bioactive glasses have generated interest in the field of bone tissue engineering due to their similarity to the natural inorganic constituent of bone. Hench and his colleagues [9,10] were the pioneers in demonstrating the suitability of bioactive glasses as bioactive, biocompatible and osteoconductive materials for bone tissue engineering. The addition of bioactive glass with alginates increases the latter’s mechanical properties [11,12]. Bioactive glasses are known to influence osteoblastic cell differentiation with an increase in the level of differentiation markers like alkaline phosphatase (ALP), osteocalcin, and osteopontin and to enhance osteogenesis through the direct control over genes that regulate cell cycle induction and progression towards a mature osteoblast phenotype [13,14,15].

In this study, we used melt-derived strontium- and zinc-containing bioactive glass in varying ratios of alginate to synthesize bone scaffolds with the use of a simple, reproducible freeze-drying technique [12]. Zinc and strontium are added to enhance the therapeutic behaviour of the glasses [16,17,18]. Zinc is not just an effective antimicrobial agent—it also stimulates osteoblast differentiation, proliferation, and mineralization through the gene expression of various proteins including type I collagen, alkaline phosphatase, and osteocalcin [17]. The composition of the glass used in this study had a higher amount of strontium as compared to ICIE16M used in our earlier study [12], as it is known that substituting strontium for calcium on a molar base enhances glass degradation and apatite formation while providing a release of strontium ions [19]. Strontium, due to its anabolic and anti-resorptive effects on bone, is already in use as strontium ranelate in the treatment of osteoporosis [18]. The local delivery of strontium ranelate in bone scaffolds avoids side-effects due to systemic intake [20]. We also added fluoride in one of the bioactive glass compositions, as it has been reported that fluoride treatment increases bone mineral density in the spine and hip areas [21].

The rationale of this study was to synthesize and characterize a biocompatible, bioactive composite material with sufficient porosity for use as a bone scaffold.

## 2. Materials and Methods

### 2.1. Glass Synthesis and Characterization

Two compositions of melt-derived glass were synthesized by mixing different proportions of analytical grade SiO_2_, P_2_O_5_, Ca_2_CO_3_, SrCO_3_, Na_2_CO_3_, K_2_CO_3_, ZnO, CaF_2_, and SrF_2_ (Sigma-Aldrich, Gillingham, UK) powders, as given in Table 1.

They were melted in a platinum-rhodium (Pt10Rh) crucible for 1 h in an electric furnace (EHF 17/3, Lenton, Hope Valley, UK). To prevent crystallization on cooling, the glasses were rapidly quenched into water. The coarse frit form of the glass was collected and dried overnight. The glass frits were milled to powder form by using a vibratory gyro-mill (Gyro mill, Glen Creston, London, UK). Later, the glass powder was sieved by using a 38 µm mesh analytical sieve (Endocotts Ltd., London, UK) [12,22]. The particle size of the bioactive glass powder was determined by using the “Master particle size analyser” (Mastersizer E), and particle size distributions are presented as D-values (D10, D50, and D90). A D-value is a diameter that, when all particles in a sample are arranged in order of ascending mass, divides the sample’s mass into specified percentages. The percentage mass below the diameter of interest is the number expressed after the “D”.

Glass powder was further characterized with the use of X-ray diffraction (XRD; X’Pert PRO, PANalytical, Cambridge, UK) to confirm the amorphous structure of the glass. The differential scanning calorimetric analysis (DSC, Stanton Redcroft DSC 1500 Rheometric Scientific, Epson, UK) of glass was used to determine the transitional temperature (Tg). Fourier transform infrared spectroscopy (FTIR spectrum GX v5.3.1)—using the software NICOLET IS10 FT-IR SPEC, Thermofisher, Waltham, MA, USA—was used to determine the structure and chemical bonds in the glass.

#### 2.1.1. Bone Scaffold Synthesis and Characterization

Sodium alginate (Fisher Scientific, Leicestershire, UK) and bioactive glass A1 were selected for the fabrication of the bone scaffold. Three types of bone scaffolds were prepared with the use of different ratios of alginate and bioactive glass (Table 2).

For the preparation of the bone scaffold, the temperature of the heat pad was set to 50 °C. A 500 mL glass beaker was placed with 100 mL of deionized water; 0.2 g of sodium lauryl sulfate (Sigma Aldrich Ltd., Gillingham, UK) was added in 100 mL of distilled water in a separate beaker and mixed well. The desired amount of alginate and bioactive glass was weighed in separate weighing boats. A thermometer was used to measure the temperature of the deionized water. When the temperature reached 50 °C, the first mixture of sodium lauryl sulfate was added, and then the alginate and bioactive glass (A_1_) was added one by one. The timer was set for 20 min, which was required for the stirring of the mixer. After 20 min, the prepared samples were poured into screw-capped bottles with a syringe. Then, the samples were quenched with liquid nitrogen for five minutes, keeping the cap open. After 5 min, the cap was screwed and the samples were transferred in the commercial freezer at a temperature of −20 °C for 48 h. The samples were divided into two groups; one group was cross-linked with 1% of w/v of calcium chloride (Sigma-Aldrich Ltd., UK), and the other was cross-linked with 1% w/v of strontium chloride (Sigma-Aldrich Ltd., UK) solution for 3 h and thereafter refrozen in a commercial freezer at −20 °C for 48 h. The previous cycle of the freeze-drying was repeated over the cross-linked samples to form a final composite bone scaffold [12,23].

The bioactivity of the scaffolds was determined by the formation of an apatite-like phase in the Tris/HCl buffer solution. The Tris/HCl buffer solution was prepared by first dissolving 15,090 g of Tris (hydroxymethyl) aminomethane (Sigma-Aldrich Ltd., UK) in 1500 mL of de-ionized water, and then 44.2 mL of 1 M hydrochloric acid (Sigma-Aldrich Ltd., UK) was added to this solution. The solution was kept in a 37 °C incubator overnight. The pH value was adjusted to 7.3 by using 1 M hydrochloric acid before diluting the solution up to a total volume of 2 L with de-ionized water. The solution was stored in a 37 °C incubator (KS 4000i control, IKA) before use [24]. For the bioactivity test, the scaffold samples were made into powder by grinding them with a mortar and pestle. The sample was dipped into the Tris/HCl buffer solution in a labelled centrifuge tube and kept in the incubator with an orbital shaker at 37 °C. The samples were left in the incubator for 15 h, 1, 3, and 7 days. Fixing the time ensured the recording of release patterns at different time points. After the desired amount of time, the samples were taken out and left alone for a few minutes to let the temperature settle down. The pH of the sample was measured with the help of the pH meter (Oaktonertech instrument, Vernon Hills, IL, USA).

After determining the pH, samples were filtered through filter paper in a centrifuge tube. The precipitates obtained after the immersion was dried in the oven at 37 °C for 48 h and analysed by X-ray diffraction (XRD), Fourier transform infrared spectroscopy (FTIR) and solid-state nuclear magnetic resonance (NMR).

The solution that was filtered through the filter paper was collected, and the releases of Ca^2+^, Sr^2+^, Na^+^, P^+5^, and Zn^2+^ in solution were quantified at different time points by using inductively coupled plasma optical emission spectroscopy (ICP-OES; Visita Pro, Varian, Inc., Oxford, UK). Calibration standards for ICP-OES analysis were prepared by using a Tris/HCl buffer solution in order to have a comparable ionic strength of calibration and sample solutions. The samples were prepared by diluting (1:5) in deionized water and adding 2% nitric acid to prevent precipitation throughout the ICP-OES test.

Scanning electron microscopy (SEM; JSM-840A, JEOL, Tokyo, Japan) was conducted on 5 mm size samples, mounted on the aluminium stub platform, and coated with gold. SEM images were analysed to determine the distribution of pores and to calculate the average pore size by using the ‘thickness’ algorithm in the ‘Bone J’ plugin for Image J [12].

Mechanical tests were carried out on scaffolds under compression mode by using an Instron MTS Bionix 100 machine. The length and diameter of the samples were measured with a digital micrometer. Tests were conducted at a crosshead speed of 2 mm/min and a load cell of 500 N by using the ‘TestWorks 4’ software. The material stiffness (Young’s modulus E) was determined from the stress-strain curve [12].

The Nuclear magnetic resonance (NMR) technique was performed to see the changes in the carbon and phosphate nuclei. The NMR experiments were performed on the 600 MHz Bruker spectrometer with the 14.1 Tesla magnetic field. The 31P MAS-NMR measurements were done at the 242.9 MHz resonance frequency of P-31 nuclei in this field by using a 4 mm sampler holder and a rotor. The 31P NMR experiments were done with a 60 s recycle delay. The spinning speed was about 9–10 kHz. The 85% solution of H_3_PO_4_ was used for referencing the chemical shift [25]. The 13C cross-polarization (CP) MAS-NMR was run at the 150.9 MHz of the resonance frequency by using the 2.5 mm zirconia rotors, which were spinning at 21 kHz with a 2 s recycle delay. The compound TMS (tetramethylsilane) was used to reference the chemical shift scale; the signal from this solution was adjusted to 0 ppm.

#### 2.1.2. Statistical Analysis

All pore sizes are expressed as mean ± standard deviation. Differences in collapse stress and Young’s modulus of the glass-reinforced and pure alginate scaffolds were assessed by using the Student’s *t*-test, where a *p-*value of less than 0.05 was considered significant.

## 3. Results and Discussion

### 3.1. Characterization of Bioactive Glass

#### 3.1.1. Particle Size Analysis

The size of the particle of the bioactive glasses was measured and shown in Table 3.The particles smaller than 38 µm were used in the fabrication of scaffolds because smaller particles provide a greater surface area of bioactive glass, providing more sites for osteoblast adhesion and osseous formation [26].

#### 3.1.2. X-Ray Diffraction Analysis (XRD)

**Figure 1:**
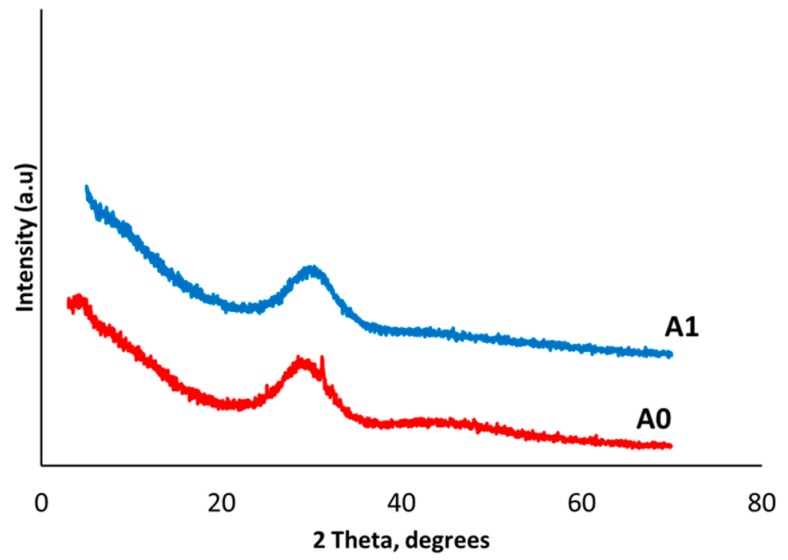
XRD graph of bioactive glasses A0 and A1.

The XRD pattern of the A0 and A1 glasses are shown in Figure 1. A1 did not have any crystallization peaks (Figure 1) and was therefore completely amorphous, which was exactly desirable. However, the A0 glass had a small peak in the graph. Amorphous glasses can produce a bioactive layer. On the other hand, crystallization reduces the bioactivity of glass [27]. A crystalline structure is responsible for a closed glass network and makes glass solid, less soluble, and less reactive. Therefore, A0 glass was not further characterized or used for the fabrication of scaffolds.

#### 3.1.3. DSC Analysis of Glass Powder

**Figure 2:**
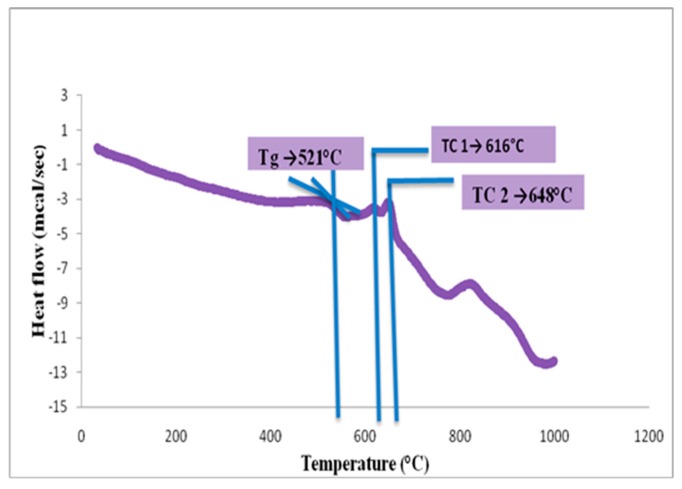
Differential scanning calorimetry (DSC) analysis of the A1 Glass.

The DSC results showed that the glass transition temperature (Tg) of the A1 glass fine particles was 521 °C, and the first (Tc1) and second (Tc2) crystallization peaks were at 616 and 648 °C, respectively (Figure 2).

#### 3.1.4. Fourier Transform Infrared (FTIR) Spectroscopy Analysis

**Figure 3:**
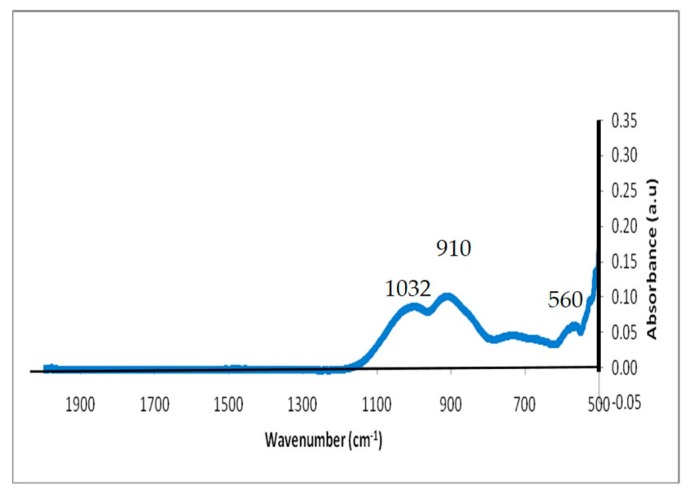
FTIR spectrum of the bioactive glass A1.

Bioactive glasses and scaffolds were also structurally characterized by FTIR to determine their chemical composition and to verify the presence of active Si–O groups in the glasses, as these groups impact the glasses’ bioactivity. Data obtained from FTIR of the glass particle showed peaks at the levels of 560, 910, and 1032 cm^−1^ (Figure 3).

The peak in the region of 870–975 cm^−1^ suggested the presence of a Si–O bond with one non-bridging oxygen per SiO4 tetrahedron [28,29]. This gives the idea that the glass contained a significant amount of the non-bridging oxygens (NBO) and was able to dissolve rapidly. Previous reports have suggested that in high phosphate-containing glasses, the peak at 500–600 cm^−1^ corresponds to the P–O bending mode as well as a Si–O–Si bending vibration in this region [30,31,32,33].

### 3.2. Characterization of Scaffolds

#### 3.2.1. FTIR Study of the Bone Scaffolds

**Figure 4:**
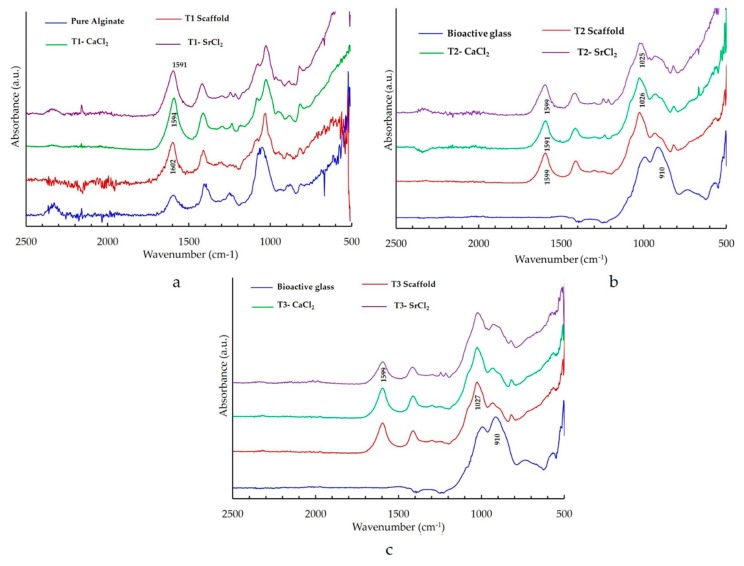
FTIR spectra showing the effect of crosslinking with calcium chloride (and strontium chloride in (**a**) type 1 (T1) bone scaffold (alginate only), (**b**) type 2 (T2) bone scaffold (alginate: bioactive glass: 1:1), and (**c**) type 3 (T3) bone scaffold (alginate: bioactive glass: 2:1).

Bone scaffolds were characterized with FTIR to ensure cross-linking after immersion in CaCl_2_ and SrCl_2_ solutions. FTIR analysis of the bone scaffolds showed the characteristic peaks of –Si–O, –OH, –COOH, and –CH. In the cross-linked samples, there was a slight shift to the right in almost all the absorbance peaks. In the type 1 bone scaffold (with no bioactive glass), the –COOH bond was seen at the level of 1602 cm^−1^ (Figure 4a). In scaffolds that were cross-linked with calcium chloride a shift to the right at 1594 cm^−1^, as well as in the scaffold that was cross-linked with strontium, a shift to the right at 1591 cm^−1^ was observed (Figure 4a). Other peaks also showed a slight shift towards the right side. The FTIR spectrum of the type 2 scaffold (with an equal quantity of bioactive glass and alginate) that was cross-linked with calcium chloride showed a shift of –COOH from 1599 to 1591 cm^−1^ (Figure 4b). The type 3 scaffold (with twice the amount of alginate) that was cross-linked with calcium chloride showed peaks at 1595 cm^−1^ and 1602 cm^−1^ (Figure 4c). Similar changes were observed in the FTIR spectrum for the scaffolds that were cross-linked with calcium and strontium.

The FTIR pattern shown in Figure 4 supports the results of previous studies [26,34], demonstrating that the scaffolds that were submerged in a crosslinking solution for 3 h underwent a remarkable physicochemical change in the scaffold, which confirmed the crosslinking of the alginate polymer chains [26,34,35]. The cross-linking occurred at the poly G, poly M, and alternating GM regions [35]. Thus, the calcium and strontium were cross-linked in the poly G, M or alternative GM regions of the alginate in the bone scaffold. As discussed in an earlier study [26], there is a slight shift in the absorbance peaks of the FTIR spectrum in a cross-linked sample. This shift occurs due to the shift of the carboxyl group. Cross-linked cations, such as calcium and strontium, interact with the –COO^−^ group of guluronic acid and mannuronic acid residue for the formation of the cross-links [35]. This cross-linking makes the scaffold insoluble in an aqueous solution [7,8,36]. Cross-linking turns the scaffold into stable hydrogels, which makes cross-linked scaffolds favourable for use as a bone material [35,36].

#### 3.2.2. Scanning Electron Microscopy Imaging of Scaffolds

**Figure 5:**
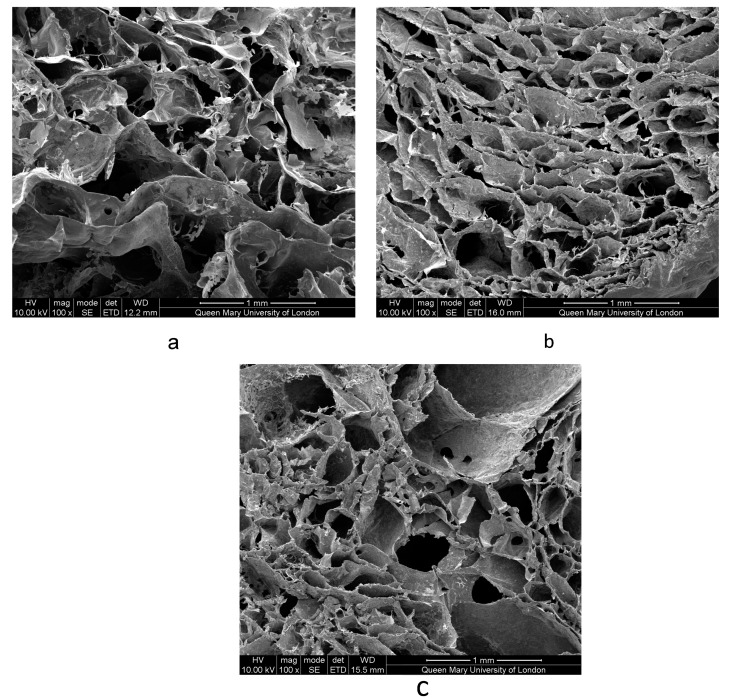
SEM image of the porous structure of bone scaffolds. Type 1 (**a**), type 2 (**b**), and type 3 (**c**) bone scaffolds. Scaffold.

The SEM images demonstrated the porous structure of all three types of scaffolds (Figure 5). Pores were interconnected and distributed within the body of the scaffolds. The average pore size for all three types of scaffolds ranged between 100 and 133 µm (see Table 4), which is adequate for the neovascularization and nutrient/metabolic waste diffusion required for new bone formation [37,38,39]. The feasible pore sizes for new bone tissue growth by tissue engineering are in the range of 100–900 μm. Outside of this range, pore sizes are too small for adequate transport over time and inhibit cell migration and neovascularization, while larger pores reduce the surface-to-volume ratios and result in slow neo tissue formation [40].

A porous structure was obtained by using the sodium lauryl sulfate as a foaming agent. Pore size was determined by using SEM and was measured with the Image J technique, but the pore size may have been affected by the binarization of the image. More accurate techniques like mercury intrusion and micro CT images can give more accurate data regarding pore size.

#### 3.2.3. Nuclear Magnetic Resonance (NMR)

**Figure 6:**
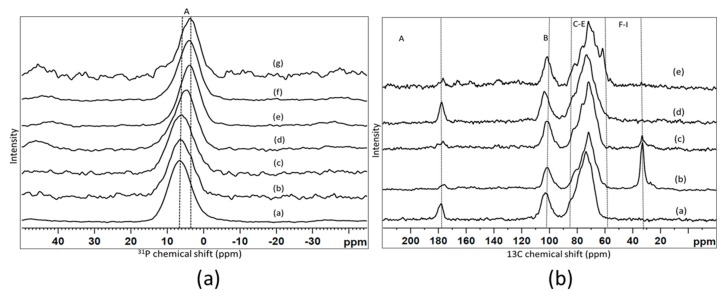
The plot (**a**) shows the 31P MAS-NMR spectrum of (**a**) bioactive glass, (**b**) type 2 scaffolds before cross-linking, (**c**) type 2 scaffolds after cross-linking with SrCl_2_ (**d**) bioactive glass after 15 h in Tris/HCl, (**e**) type 3 scaffolds cross-linked with SrCl_2_ after 15 hrs in a Tris/HCl buffer, (**f**) type 2 scaffolds cross-linked with CaCl_2_ after 15 hrs in Tris/HCl, and (**g**) type 2 scaffolds cross-linked with CaCl_2_ after two weeks in Tris/HCl. The plot (**b**) shows 13C cross-polarization (CP)-MAS-NMR spectra for (**a**) sodium alginate, (**b**) type 1 scaffolds after crosslinking with SrCl2, (**c**) type 3 scaffolds cross-linked with CaCl_2_, (**d**) type 3 scaffolds cross-linked with SrCl_2_ after 15 hrs in a Tris/HCl buffer, (**e**) type 3 scaffolds cross-linked with SrCl_2_ after two weeks in a Tris/HCl buffer. The capital letters refer to the resonance assignments listed out in Table 5 and Table 6.

Figure 6a shows the 31P MAS-NMR spectra for the selected composite scaffold samples in comparison with the initial glass powder, before and after immersion into the Tris/HCl buffer solution. All the spectra showed one broad unresolved signal that corresponded to an amorphous orthophosphate, with the position varied slightly from sample to sample [41]. The peak positions extracted from the spectra are presented in Table 5. We can see that there were no changes in the spectra of the cross-linked scaffold compare to the initial glass powder, which implies that no changes occurred in the environment of the phosphorus atoms in the composite scaffold compared to the glass. The peak position of the signal clearly changed after immersion into the Tris/HCl buffer and became less positive. This could have been due to a partial dissolution of glass during the immersion. The changes were most probably due to Na^+^ and K^+^ ions being released from the glass and the replacement of those cations with the available Ca^2+^ and Sr^2+^ to balance the charge of the orthophosphate. Most of the changes occurred after the first few hours (15 h), and no changes were observed for much longer durations (two weeks).

Figure 6b displays the 13C CP-MAS-NMR spectra of two types of scaffold samples that were immersed for various durations, and these are compared with the initial sodium alginate powder. All the spectra consist of three regions, according to the literature [42]. The signals denoted A, B and the C–H group of signals. The peak positions identified from the spectra with the assignment are given in Table 6. The region/signal between 170 and 180 ppm ‘A’ belonged to the carboxylic carbon atoms. The region/signal between 90 and 110 ppm ‘B’ corresponded to the anomeric region (Figure 6). Multiple signals from 90 to 60 ppm were assigned to the pyranose region, and the corresponding assignments are plotted in Table 6. After cross-linking in a CaCl_2_ solution, a signal was observed at about 33 ppm, which could have been assigned to an alkyl chain, cross-linked scaffold before and after immersion into the Tris/HCl buffer. In addition, there was some intensity at about 61 ppm (signal H), especially clear on the spectrum of the sample immersed for two weeks. The accurate assignment of this particular signal has to be further clarified. A slight shift between the peak position in the cross-linked scaffold compared to the non-cross-linked sodium alginate was also observed previously [12,23,42].

The 31P MAS-NMR (Figure 6a) showed that phosphorus existed as an orthophosphate in the bioactive glass and should favour glass bioactivity. The solid-state NMR result also gave evidence for slight changes in the scaffold after the cross-linking. The phosphorus environment showed small changes. More changes were observed in the carbon spectrum (Figure 6b), including the peak positions, which were similar to an earlier study [42].

#### 3.2.4. Compression Testing of Scaffolds

**Figure 7:**
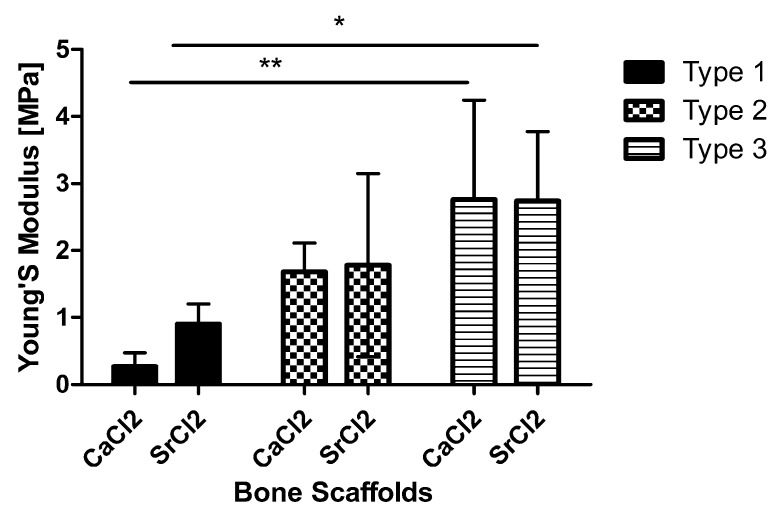
The material stiffness of the three types of bone scaffolds cross-linked with CaCl_2_ and SrCl_2_. * *p* < 0.05 and ** *p* < 0.01.

The average Young’s modulus was calculated from the stress-strain curves obtained from all the samples. The bone scaffold that was made with more alginate and bioactive glass (type 3 scaffolds) demonstrated better mechanical properties in compression as compared to other scaffolds. This change was statistically significant at *p* < 0.001 and *p* < 0.05 when comparing type 1 (pure alginate scaffolds) with type 3 scaffolds cross-linked with CaCl_2_ and SrCl_2_, respectively (Figure 7). The average Young modulus values calculated for type 3 scaffolds that were cross-linked with CaCl_2_ and SrCl_2_, respectively, were 2.8 ± 1.5 and 2.74 ± 0.9 MPa as compared to 1.68 ± 0.4 and 1.8 ± 1.3 Mpa for type 2 bone scaffolds with equal quantity of glass and alginate ration. The cross-linking agent had no overall statistically significant effect on the material stiffness in compression. Half of the samples obtained from the type 1 scaffolds that contained the only alginate could not withstand the compression test and were crushed immediately. The limited data obtained for type 1 scaffolds showed an average Young’s modulus of 0.2 ± 0.2 and 0.9 ± 0.3 Mpa for samples cross-linked in CaCl_2_ and SrCl_2_, respectively.

The mechanical testing results of the type 1 scaffold, which was made only with alginate, matched with the results of a previous study [43]. The incorporation of the bioactive glass with the alginate improved the compressive strength of the scaffold. In this project, the bioactive glass that was used had a higher amount of strontium as compared to our earlier study [12], and cross-linking with the strontium was done to improve the compressive strength; we were able to improve the glass’s mechanical properties, as measured by the Youngs modulus of the composite bone scaffolds from 1.83–0.66 MPa, the value reported in our earlier study, to 2.8 ± 1.5 MPa. However, this value can be further improved to match bone mechanical properties. Moreover, more experiments can be carried out by using various types of alginates with varying compositions of M- and G-blocks that will affect the mechanical properties of the alginates and the scaffolds [44].

### 3.3. Tris-Buffer Study

#### 3.3.1. pH Measurements

**Figure 8:**
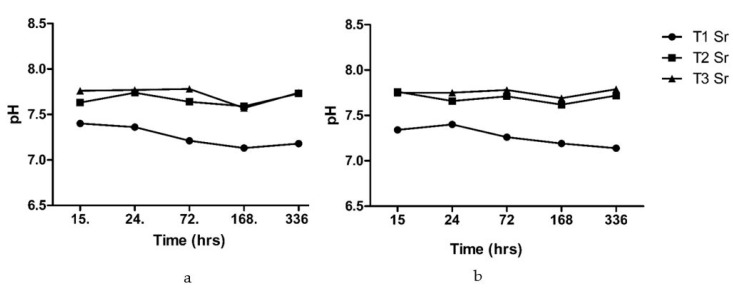
The changes in the pH after the immersion of T1 (pure alginate), T2 (alginate and bioactive glass 1:1), and T3 (alginate and bioactive glass 2:1) scaffolds in the Tris-buffer solution cross-linked with (**a**) calcium chloride and (**b**) strontium chloride.

The pH was measured for the bone scaffold after immersion in the Tris-buffer at different time points (Figure 8). The changes in pH were observed in the range of 7.13–7.4 for the type 1 pure alginate scaffolds and 7.62–7.76 for the composite scaffolds. The composite scaffolds showed a slight increase in the pH level at first, followed by a dip, and then it maintains the alkaline pH above 7.7.

A study of pH changes of the scaffold upon immersion into the buffer solution was performed because it was a fast and simple test regarding how the bioactive glass or composite based on bioactive glass would behave in vitro and in vivo [41]. The initial increase observed in the pH of the scaffolds with bioactive glass was due to the release of ions from the bioactive glass. During the healing phase, the normal serum pH decreased, presumably due to the accumulation of the acidic metabolites in tissue fluids. Subsequently, the alkaline pH favoured the deposition of the mineral content. The pH plays a regulatory role in the healing and mineralization of defect areas [45].

The pH analysis of the bone scaffold after immersion in the Tris-buffer showed a neutral to slightly alkaline pH. The scaffold with only the alginate showed a slight decrease in the pH, whereas the scaffold with alginate and bioactive glass in the same ratio and the scaffold with alginate and bioactive glass with a ratio of 2:1 showed pH values in the range of the alkalinity. The alkaline pH allowed the bone-forming cell to form a new bone matrix. The increase in the pH in the early period was due to the exchange of the Na+/H+ ions. The Na^+^ or Ca^2+^ near the surface of the glass went into the solution, resulting in a pH rise. This evidence is supported by the release profile of the sodium ion, as they showed initial increased release rates from the scaffold. The initial changes were then maintained for the next two weeks. The bone scaffold with alginate and bioactive glass could maintain the alkaline media that are required for the growth of a new bone matrix [45].

#### 3.3.2. Degradation Study Using Inductive Coupled Plasma-Optical Emission Spectrometry (ICP-OES)

**Figure 9:**
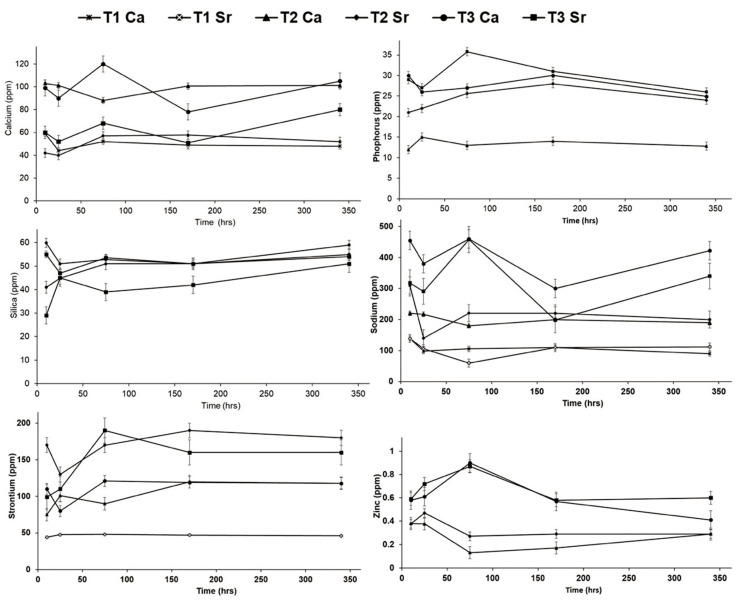
The release of ions from the bone scaffold after immersion in the Tris-buffer.

The release of ions from the scaffold after immersion in the Tris-buffer was quantified by the ICP-OES, and the release profile for calcium, phosphorus, sodium, zinc, and strontium ions are shown in Figure 9. The scaffolds were able to release ions, and the maximum release of ions was shown by the composite scaffolds.

The release of calcium from the T2 and T3 composite scaffolds that were cross-linked with calcium chloride was highest. This indicates that the amount of Ca^2+^ release was dependent on the composition of bioactive glass and the cross-linking agent. The trend of release seemed to be stabilized after about one week and was maintained even after two weeks. A similar trend was observed for phosphorus, zinc and strontium ions, with the maximum release shown by the type 3 composite scaffolds that were linked with strontium chloride. The release of silicon ions at about 15 h was highest for the type 3 scaffolds, but at the end of two weeks, the level of the released silicon from the different scaffolds was almost at the same point.

The release of these ions from bioactive glasses has stimulating effects on osteoblasts, regulating the expression of several genes such as key osteoblastic markers and extracellular matrix proteins [16,17,18]. A recent study demonstrated that silicon-containing biomaterials are also excellent at promoting angiogenic events, such as endothelial (progenitor) cell homing, cell polarization, migration, angiogenic differentiation, and neo-blood vessel sprouting [46].

### 3.4. Characterization of Scaffolds after Immersion in Tris-Buffer

#### 3.4.1. Fourier Transform Infrared Spectroscopy Analysis

**Figure 10:**
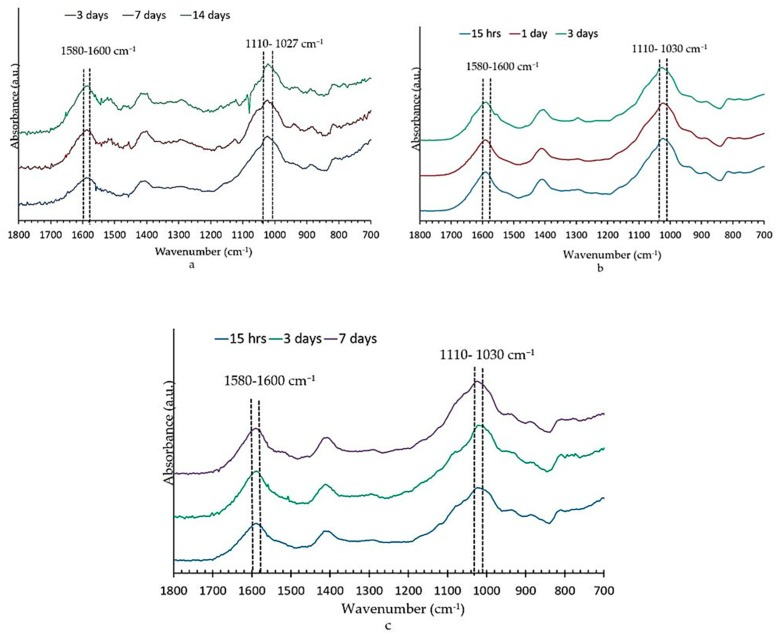
FTIR spectra of type 1 (**a**), type 2 (**b**), and type 3 (**c**) bone scaffolds that were cross-linked with calcium chloride (**a**,**b**) and strontium chloride (**c**) after immersion in the Tris/HCl buffer at different time points.

FTIR was performed on all three types of scaffolds after they were removed from the Tris-buffer to evaluate the apatite formation. No change was observed in the samples that were cross-linked with strontium chloride or calcium chloride. The FTIR spectra from type 1 and 2 samples that were cross-linked with calcium chloride and the type 3 samples that were cross-linked with strontium chloride are shown in Figure 10. The change in the type 1 scaffold after immersion in the Tris-buffer was not much, and changes tended to occur within the first 15 h and then maintained that change up to two weeks for all the scaffolds.

Recent reports about strontium-containing bioactive glasses also showed that bands at 560 cm^−1^ and 610 cm^−1^ and 1032 cm^−1^ are characteristic of apatite formation [24]. In this study, the noise level at this area was more in the spectrum, and well-defined peaks were not evident in this range of the spectrum for any of the scaffolds and are therefore not shown in Figure 10. The absence of characteristic split bands at 560 and 600 cm^−1^ may have been a result of a mixed Ca/Sr- hydroxy-carbonate apatite being formed, and the disordered structure of such apatite may have led to the broadening of the phosphate vibrational mode and the loss of this characteristic peaks.

The non-bridging oxygen Si–O− band at 910–920 cm^−1^ disappeared upon immersion into the Tris-buffer. New bands appeared at about 790 cm^−1^, and we assigned this to the Si–O–Si bond vibration between two adjacent SiO4 tetrahedra. These changes indicated the formation of a silica-gel surface layer after the leaching of Ca^2+^and Na^+^ and the formation of Si–OH bonds. 

All the spectra (Figure 10) clearly showed the sharpening of a band at about 1110–1030 cm^−1^ that is associated with both Si–O–Si and crystalline orthophosphate [40]. The band at 1020 cm^−1^ was assigned to HPO_4_^2−^ in the crystalline apatite, and the band at 1110 cm^−1^ was assigned to ν1 CO_3_^2−^ at the substitution site A [47].

The FTIR spectrum (Figure 10) of all the scaffolds showed the carbonate absorption bands at 1580–1600 cm^−1^. Peaks were more prominent in the composite scaffolds, indicating that carbonate anions had been substituted for certain phosphate positions in the apatite lattice (B-type substitution). In precipitated biological apatite, the predominant substitution appears to be CO_3_^2−^ anions for PO_4_^3−^ anions, but, usually, PO_4_^3−^ can replace small amounts of OH^−^ ions (A-type substitution) [47].

#### 3.4.2. Scanning Electron Microscopy

**Figure 11:**
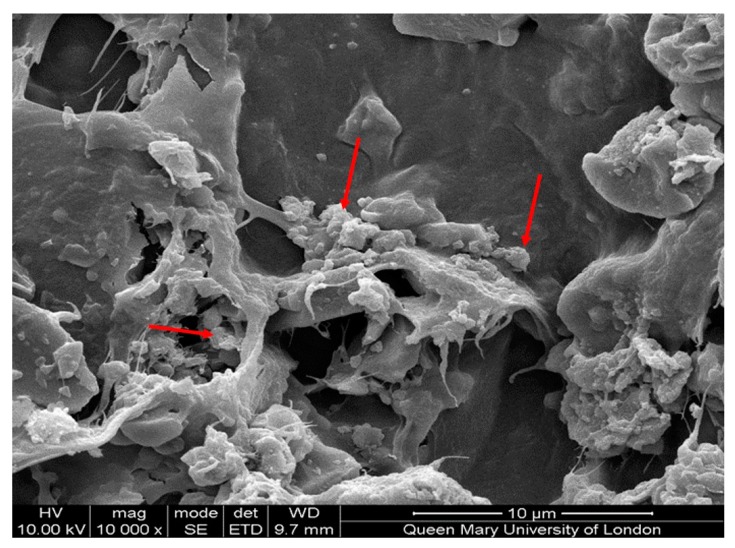
The SEM image of the calcium orthophosphate precipitation on the scaffold after the bioactivity test is shown by arrows.

The SEM results (Figure 11) suggested some kind of precipitation on the surface of the scaffold. Along with other experimental data, it can be said that this precipitation was the resultant orthophosphate, which formed as a result of the release of ions from the bone scaffold. Typical needle-shaped hydroxyapatite crystals were not found on the surface of the scaffolds.

#### 3.4.3. Nuclear Magnetic Resonance (NMR) of Scaffolds after Immersion in Tris-Buffer

The NMR result (Figure 6a) also showed the unresolved signal for the calcium orthophosphate Ca_8_(PO_4_)6H_2_5H_2_O (OCP) [48].

An earlier study showed [49] that immersion of the scaffolds based on glass ceramics in a Tris-buffer solution promoted faster degradation. Bioactive glasses with a high phosphate content after immersion in a Tris-buffer favour the formation of an octacalcium phosphate (OCP) precursor phase that subsequently transforms into apatite [50,51,52]. However, while designing bioactive glasses, it is important to consider that, above a certain concentration of Sr or Sr/Ca ratio, the octacalcium phosphate precursor phase is unable to form, ultimately retarding the formation of a hydroxycarbonate-like phase. Moreover, the formation of OCP is generally favoured by less basic pHs, as reported in this study, and higher P:Ca ratios [53]. A high pH > 9 promotes the direct formation of hydroxyapatite. 

Further studies are underway in our laboratory to synthesize scaffolds with improved bioactivity by using bioactive glasses with varying amounts of the concentration of strontium and phosphorus with alginates.

## 4. Conclusions

A porous scaffold that was created by using strontium- and zinc-containing bioactive glass in combination with alginate was successfully synthesized. Upon immersion in a Tris/HCl buffer solution, the scaffolds were able to release strontium and zinc ions, and an orthophosphate layer was formed on the surface of the scaffold; this was supported by FTIR, NMR, and ICP-OES results. The addition of strontium in the bioactive glass enhanced the mechanical properties of the scaffolds but needs to be further improved to match bone properties. A cell culture study followed by cytotoxic and osteogenic differentiation assays is required before the in vivo implantation of these scaffolds.

## Figures and Tables

**Table 1:** The composition of the bioactive glass (mol.%).

	SiO_2_	P_2_O_5_	CaO	CaF_2_	SrO	SrF_2_	Na_2_O	K_2_O	ZnO	Melting Temperature (°C)
A0	36.4	6.0	26.5	2.2	26.5	2.2	0	0	0	1500
A1	44.0	5.0	15.0	0	15.0	0	10.0	10.0	1.0	1420

**Table 2:** Showing the weight ratio of alginate and bioactive glass (BG) A1 in three types of bone scaffolds.

Bone Scaffold	Alginate: BG	Mass of Alginate (g)	Mass of BG (g)
Type 1 (T1)	1:0	3	0
Type 2 (T2)	1:1	3	3
Type 3 (T3)	2:1	6	3

**Table 3:** Particle size analysis of the glass.

% of Particles	Particle Size <38 (µm)	Particle Size >38 (µm)
D(v,0.9) (90%)	13.74	65.75
D(v,0.1) (10%)	1.21	1.51
D(v0.5) (50%)	4.20	22.06

**Table 4:** Pore size of the scaffold expressed as mean ± S.D.

Sample Type	Pore Size (µm)
(T1) Scaffold (only alginate)	133 ± 10.5
(T2) Scaffold (alginate and bioactive glass 1:1)	100 ± 8.5
(T3) Scaffold (alginate and bioactive glass 2:1)	107 ± 8.1

**Table 5:** Assignments of the resonances in the 31P MAS-NMR spectrum (ppm).

	Sample Name	31P Chemical Shift at A Region (ppm)
(a)	Bio glass	6.6
(b)	Type 2 before cross linking	6.5
(c)	Type 2 after cross linking with SrC_l2_	6.0
(d)	Glass after 15 hrs in Tris/HCl	4.7
(e)	Type 2 Srcl_2_ after 15 hrs in Tris/HCl	3.8
(f)	Type 2 CaCl_2_ after 15 hrs in Tris/HCl	3.9
(g)	Type 2 CaCl_2_ after 14 days in Tris/HCl	3.8

**Table 6:** Assignments of the resonances in the 13C CP-MAS-NMR spectrum (ppm).

	Sample Name	13C Chemical Shift at Region (ppm)								
		A	B	C	D	E	F	G	H	I
(a)	Sodium alginate	178	102.98	84	78	71				
(b)	Type 1after cross linking with SrCl_2_	175.8	101.3	81	76	71.8				33
(c)	Type 3 after cross linking with Cacl_2_	176.7	102	83	76.3	71.8				33.2
(d)	Type 3 cross-linked with SrCl_2_ after 15 hrs in Tris	177.7	103.7	82	77	73.4				
(e)	Type 3 cross-linked with SrCl_2_ after 14 days in Tris	176.8	101. 8	81.9	76.3	72	68.6	66.2	61.7	
